# A Case Follow-Up Report: Possible Health Benefits of Extra Virgin Olive Oil

**DOI:** 10.1100/tsw.2004.141

**Published:** 2004-09-24

**Authors:** Said Shahtahmasebi, Shahnaz Shahtahmasebi

**Affiliations:** RaDiSol, (R & D Integrated Solutions), Christchurch, New Zealand; ^2^ Consultant Dietician, Wagga Wagga, Australia

**Keywords:** diet, nutrition, olive oil, coronary heart disease, cholesterol, public health

## Abstract

In the course of a case study, a number of issues regarding the dynamics of blood cholesterol levels were identified. In this follow-up report, these issues are addressed. For example, issues of past behaviour and seasonality, intraindividual variation, and nonstationarity appear important over and above controllable variables such as diet and exercise. In this report, we conceptualise an alternative protective role for the dynamic blood cholesterol levels in a healthy population. Furthermore, regular consumption of extra virgin olive oil as produced in this case study may interact with the dynamics of cholesterol naturally. We recommend that future studies of this kind ought to include a time series of blood cholesterol based on daily measurements or intervals much shorter than the bimonthly measurements and to include measures of overall well being as covariates.

## INTRODUCTION

Currently much of the medical advice and policies on the prevention of heart disease is based on an apparent relationship between cholesterol levels and coronary heart disease (CHD) and, in turn, between unsaturated oils and cholesterol levels[1,2,3]. For example, expert reports from WHO (http://www.who.int/hpr/NPH/docs/gs_chronic_disease.pdf, http://www.who.int/mediacentre/releases/2003/pr20/en/) and the American Medical Association (http://www.ama-assn.org/ama/pub/printcat/11004.html) have recommended the increased consumption of fruit, vegetables, and unsaturated oils. There is some evidence to suggest that unsaturated oils that have retained antioxidant agents may provide a health benefit in terms of a lowered cholesterol level[4].

On the other hand, a brief review of the recent literature suggests that cholesterol levels may be a dynamic process outcome. For example, studies of blood cholesterol levels suggest a significant seasonal effect[5,6,7,8,9,10,11,12,13,14], individual specific (intraindividual) effect[9,10], stress[15], physical activity[16], sunlight or natural bright light, climatic changes[17,18], low cholesterol levels, and depression and suicide[19,20].

Therefore, to tease out the net effect of the extra virgin olive oil on blood cholesterol levels, a longitudinal trial of a cohort of healthy individuals is necessary. In the absence of such trials, we followed up the case reported earlier[4] to gain some insight into the dynamics of blood cholesterol. The follow-up case study is intended to raise some issues of interest to further stimulate international debate and cooperation.

## CASE REPORT: FOLLOW-UP

Briefly, the case is a male individual in his mid-forties, physically active with stable dietary habits, and no history of major health problems or illnesses requiring a blood test for cholesterol levels. One of the issues raised in the case report[4] was that we had no knowledge of the dynamics of blood cholesterol. Having observed the case for some time, it transpired that life events over a period of time may provide natural experimental units. Naturally, it was decided to utilise such a phenomena to our advantage similar to a quasi-experimental design type study. For example, the case went through a phase (as we all may do), where physical activities were reduced to a minimum (walking about 3 km once/twice a week). We asked the case to note when such a change occurred and to make the phase last until the next blood test. In order to make this possible, we chose monthly intervals for the blood tests. The person had no problems with this plan and agreed to be observed further over a short period of time.

Before the observations began, the person took part in an exercise ECG. The exercise was carried out primarily to ensure a healthy heart to minimise the health risks to the case due to the experiment. The results showed no signs of heart problems. For consistency, we arranged all the blood tests through the same laboratory. The laboratory operates a first-come-first-served system and no appointments were necessary. Blood samples were taken in the morning between 9 and 9.30 AM by a qualified nurse at a designated branch of the laboratory and sent off for lipid analysis. Fasting was observed for all blood tests. The results were received by post the following day. Full blood cholesterol levels are reported in Table 1.

**TABLE 1**
**Details of Blood Cholesterol Tests Over Several Time Points**

| Cholesterol (mmol/l) | HDL (> 1)* | LDL (< 3.5)* | Triglyceride (< 2)* | Total (3–5)* |
|---|---|---|---|---|
| Baseline (19/2/2002) | 1.8 | 3.8 | 0.7 | 5.8 |
| Follow-up (30/7/2003) | 1.8 | 3.4 | 0.5 | 5.4 |
| | | **Case Follow-up 2004** | | |
| Follow-up (04/02/04) | 1.7 | 4.3 | 0.7 | 6.3 |
| Follow-up (09/03/04) | 1.9 | 4.1 | 0.6 | 6.3 |
| Follow-up (14/04/04) | 1.9 | 3.4 | 0.6 | 5.6 |
| Follow-up (14/05/04) | 1.8 | 4.5 | 0.5 | 6.5 |
| Follow-up (18/05/04) | 2 | 4.8 | 0.6 | 7.1 |
| Follow-up (19/05/04) | 1.7 | 4.3 | 0.6 | 6.3 |
| Follow-up (24/05/04) | 1.9 | 5.0 | 0.7 | 7.2 |
| Self-reported (23/06/04) | 2.1 | 4.3 | 0.6 | 6.7 |

* New Zealand National Heart Foundation Recommended Ranges.

**Period to 4/2/04:** The person had consistently used extra virgin olive oil in cooking. However, during December 2003 and over the Christmas period (2003), as expected, the dietary pattern changed in line with the festive season and the consumption of extra virgin olive oil was considerably reduced to 1 l for the whole of December 2003 and January 2004. The case had also ceased all physical exercise. We deferred the blood test to the end of January to allow the adjustment for the Christmas period. The second follow-up of lipid analysis was carried out on 4 February 2004, which suggested an increase in the blood cholesterol. Since the first follow-up, the total cholesterol had increased from 5.4 to 6.3; LDL and triglyceride had increased whilst HDL had slightly decreased, see Table 1.

**Period 4/2/04 to 9/3/04:** During this period, the person was not ready to begin his exercise routine, but the consumption of extra virgin olive oil had returned to its usual levels (about 1 l per 10 days). No other changes were noted during this period. The third follow-up lipid analysis was carried out on 9 March 2004. There was no change in the total cholesterol. It is interesting to note, however, that both LDL and triglyceride had decreased whilst HDL had increased.

**Period 9/3/04 to 14/4/04:** The person was still not ready to start his exercise routine. For the next 4 weeks until the next blood test, the case agreed to try a natural food item that was produced by combining a variety of natural food ingredients with extra virgin olive oil (such as garlic, fresh herbs, and lemon juice). The plan involved the daily consumption of one spoonful of the oil-based mixture in any way the case preferred, e.g., as a salad dressing, mixed with sandwich fillings, poured over cooked food, or simply taken with bread. No other changes were noted over the next 4 weeks. The fourth follow-up of lipid analysis was carried out 14 April 2004. As can be seen from Table 1, the total cholesterol had dropped from 6.3 to 5.6 with no change in HDL and triglyceride whilst LDL had decreased.

**Period 14/4/04 to 14/5/04:** The case decided to get back into his exercise routine. He started with regular jogging three to four times a week averaging about 15 km per week. With the start of the exercise routine, the consumption of the oil-based mixture was substantially reduced and ceased within the first week. No other changes were observed. During this period, we expected a change in the total cholesterol level. Indeed, the results from the monthly test showed that total cholesterol and LDL had increased to 6.5 and 4.5, respectively, with minor changes in HDL and triglyceride. The test had been carried out on the Friday (14/05/04) and the results had become known the following Monday.

**Period 14/05/04 to 24/05/04:** The 16% increase in total cholesterol prompted us to reintroduce the daily consumption of the remainder of the oil-based mixture back into the case's diet (17/5/05). The mixture was used twice daily and was finished by 21/5/05. We also decided to take another sample soon after the previous test rather than wait until the next test, a common practice in the application of SPC (Statistical Process Control). This test, carried out on 18/5/04, showed further elevation of cholesterol (a 27% increase since the test on 14/5/04).

Given the daily variation in the cholesterol levels, it is not easy for us to isolate the net effect of the oil-based mixture on the cholesterol levels from other factors. However, we were rather interested in the results of the follow-ups 14/05/04 and 18/05/04, as, apart from the introduction of exercise in place of the oil-based mixture, no major changes in the diet had occurred. It is plausible that the dynamics of blood cholesterol and how this relates to the individuals' overall health may explain some of the variation. We considered the seasonal effect (see introduction) as part explanation, however, the seasonal effect did not appear to be present in the baseline (mid-summer) and the first follow-up (mid-winter). So we went back to the case and asked him to recall and describe his overall health for the period of experimentation and the week preceding the test 14/05/04. The case described a depressed episode (of feeling low, feeling the blues), which had started a week earlier (without any identifiable cause) and was over by the 17/05/04. So with the case's consent, another test was carried out the next day (19/5/05). It is interesting to note the decrease in total cholesterol (11%), LDL (10%), and HDL (15%) and no change in triglyceride over night. The case had been consuming the oil-based mixture since 17/05/04. If the regular consumption of the oil-based mixture is maintained, we expect that the cholesterol levels of the case to fluctuate daily over the short period but having a downward trend over a longer period. The next day after the blood test on 19/5/04, the case expressed the onset of another episode of feeling low and could not explain why. We cannot be certain if feeling "the blues" might be the result of a rapid reaction to lowered cholesterol levels. However, the case reported that the short episode was over by Sunday 23/5/04. This was an opportunity we could not ignore; the case agreed to another blood test the following Monday (24/5/04) by which time the case reported feeling his normal self. However, at first we expected a decrease in total cholesterol, but following his comments about feeling his normal self, we predicted elevated cholesterol levels and changed our expectation. Indeed, the results showed increases in total cholesterol (14%), HDL (12%), LDL (16%), and triglyceride (17%).

**Period 24/5/04 to 23/6/04:** The case further described a year of sustained and long periods of work-related stress that had negatively affected his mental well being. Furthermore, the case had developed a muscular pain and was advised by his GP to stop physical activities for a month. We decided to stop the experiment. The case decided to continue with a small daily dose of the mixture, which he prepared himself and reported 7 and 14% decrease in the total cholesterol and LDL, respectively, and 10% increase in HDL in a final blood test taken on 23/6/04. He also reported no incidence of “feeling the blues” during this period.

## DISCUSSION

It is not possible to disentangle the systematic from random variation without a properly designed study as to how cholesterol levels, coronary heart disease (CHD), suicide and depression, and individual and environmental parameters are inter-related. Nevertheless, nutritional interventions of this sort may be useful. Quite apart from the fact that the ingredients are natural and that there is no processing (cooking/heating or artificial additives) involved, the intervention is cheap and accessible to the wider population. Such interventions could be viewed as a natural cholesterol regulator and more of a preventative measure. Future studies need to include an economic evaluation of the overall long-term benefits of such prevention/intervention and consideration to the dynamics of blood cholesterol.

Some questions relating to the dynamics of blood cholesterol levels arise from this case study; specifically:

- Could, the extra intake of extra virgin olive oil in the period to 09/03/04 be held responsible for capping the cholesterol levels? (Controllable factors)
- Do cholesterol levels vary daily? (Time series/temporal dependence)
- All variation in blood cholesterol is not accounted for by controllable factors; could individual specific characteristics also explain some of the variation? (Intraindividual variation/heterogeneity)
- Do cholesterol levels have a memory? (Past behaviour)
- Do cholesterol levels respond to external factors? (Nonstationarity/external changes)

These questions cannot be addressed with this case study, however, we may seek guidance from the literature. The literature reports the protective benefits of extra virgin olive oil, which was discussed earlier[4]. Larger studies suggest that there are controllable factors such as diet and exercise, as well as intraindividual and daily variation (e.g., see [9]) that may be affecting cholesterol levels. On the other hand, some other authors report that changes in blood cholesterol may occur independent of diet and possibly due to external factors such as seasonal and environmental changes[5,6,7,8,9,10,11,12]. “Stress” has also been reported to be responsible for raised blood cholesterol levels[15]. On the other hand, whilst depression and suicide have been associated with low cholesterol levels[19,20], studies of depression and suicide also report a seasonality effect in the outcomes[13,14,17,18]. There appears to be a difference of opinion in the peak trough of the seasonal effect between studies. For example, some authors have suggested alternative seasons for the peak trough; e.g., the end of spring as the peak for suicide[13], spring and autumn for the highest and lowest concentration of serum cholesterol[5], spring and summer for the highest and lowest concentration of cholesterol levels[11,12], and that seasonal mood variations or seasonal affective disorder (SAD) may not be due to a differential pattern in bright light exposure[21,22].

Thus, studies of seasonality in cholesterol levels have concentrated on seasonal parameters such as exposure to light, climatic differences between seasons, and so on. However, some studies suggest that depression is much more common in the population than has been reported[23,24]. Therefore, it is plausible that in a time series based on daily (as opposed to bimonthly) variation in cholesterol levels, we may observe a “seasonality” effect during a specific period. Given the case’s accounts in this case study of his raised cholesterol levels coinciding with the ending of a depressed period, the question that arises is what might be the possible health consequences to the healthy population if cholesterol levels did not respond to stress?

Clearly, the implication for practice cannot be ignored, for example a recent study[10] suggests that some individuals may have been mistakenly diagnosed as having high cholesterol levels, whilst another[5] found no direct relationship between seasonal variation in serum cholesterol and seasonal variation in either the incidence of or mortality due to ischaemic heart disease. It is, therefore, plausible that, at least in part, the variation in cholesterol levels may well be due to an individual-specific latent trait to maintain overall well being. We recommend a major review of the literature on this topic to inform future study designs in addressing the relevant issues.

## References

1. Kelly, C.M., Smith, R.D., and Williams, C.M. (2001) Dietary monounsaturated fatty acids and haemostasis. *Proc. Nutr. Soc.* **60(2),** 161–170.
2. Jossa, F. and Mancini, M. (1996) [The Mediterranean diet in the prevention of arteriosclerosis]. *Recenti Prog. Med.* **87(4)**, 175–181.
3. Kushi, L.H., Lenart, E.B., and Willett, W.C. (1995) Health implications of Mediterranean diets in light of contemporary knowledge. 2. Meat, wine, fats, and oils. *Am. J. Clin. Nutr.* **61(6 Suppl)**, 1416S–1427S.
4. Shahtahmasebi, S. and Shahtahmasebi, S. (2003) A case report of possible health benefits of extra virgin olive oil. *TheScientificWorldJOURNAL* **3**, 1265–1271.
5. Fyfe, T., Dunnigan, M., Hamilton, E., and Rae, R. (1968) Seasonal variation in serum lipids, and incidence and mortality of ischaemic heart disease. *J. Atheroscler. Res.* **8**, 591–596.
6. Thomas, C.B., Holljes, H.W.D., and Eisenberg, F.F. (1961) Observations on seasonal variations in total serum cholesterol level among healthy young prisoners. *Ann. Intern. Med.* **54**, 413–430.
7. Warnick, R.G. and Albers, J.J. (1976) Physiological and analytical variation in cholesterol and triglycerides. *Lipids* **11**, 203–208.
8. Gordon, D.J., Trost, D.C., Hyde, J., Whaley, F.S., Hannan, P.J., Jacobs, D.R.J., et al. (1987) Seasonal cholesterol cycles: the Lipid Research Clinics Coronary Primary Prevention Trial placebo group. *Circulation* **76(6)**, 1224–1231.
9. Bluher, M., Hentschel, B., Fauci, R., and Richter, V. (2001) Influence of dietary intake and physical activity on annual rhythm of human blood cholesterol concentrations. *Chronobiol. Int.* **18(3)**, 541–557.
10. Ockene, I.S., Chiriboga, D.E., Stanek, III, E.J., Harmatz, M.G., Nicolosi, R., Saperia, G., et al. (2004) Seasonal variation in serum cholesterol levels: treatment implications and possible mechanisms. *Arch. Intern. Med.* **164(8)**, 863–870.
11. Kristal-Boneh, E., Harari, G., and Green, M. (1993) Circannual variations in blood cholesterol levels. *Chronobiol. Int.* **10**, 37–42.
12. Harlap, S., Kark, J., Baras, M., Eisenberg, S., and Stein, Y. (1982) Seasonal changes in plasma lipid and lipoprotein levels in Jerusalam. *Isr. J. Med. Sci* **18**, 1158–1165.
13. Granberg, D. and Westerberg, C. (1999) On abandoning life when it is least difficult. *Soc. Biol.* **46(1–2)**, 154–162.
14. Maes, M., De Meyer, F., Peeters, D., Meltzer, H., Cosyns, P., and Schotte, C. (1992) Seasonal variation and meteotropism in various self-rated psychological and physiological features of a normal couple. *Int. J. Biometeorol.* **36(4)**, 195–200.
15. Stoney, C., Niaura, R., Bausserman, L., and Matacin, M. (1999) Lipid reactivity to stress. I. Comparison of chronic and acute stress responses in middle-ages airline pilots. *Health Psychol.* **18**, 241–250.
16. Leppamaki, S.J., Partonen, T.T., Hurme, J., Haukka, J.K., and Lonnqvist, J.K. (2002) Randomized trial of the efficacy of bright-light exposure and aerobic exercise on depressive symptoms and serum lipids. *J. Clin. Psychiatr.* **63(4)**, 316–321.
17. Doganay, Z., Sunter, A.T., Guz, H., Ozkan, A., Altintop, L., Kati, C., et al. (2003) Climatic and diurnal variation in suicide attempts in the ED. *Am. J. Emerg. Med.* **21(4)**, 271–275.
18. Molin, J., Mellerup, E., Bolwig, T., Scheike, T., and Dam, H. (1996) The influence of climate on development of winter depression. *J. Affect. Disord.* **37(2–3)**, 151–155.

19. Ainiyet, J. and Rybakowski, J. (1996) [Low concentration level of total serum cholesterol as a risk factor for suicidal and aggressive behavior]. *Psychiatr. Pol.* **30(3)**, 499–509.
20. Brunner, J., Parhofer, K.G., Schwandt, P., and Bronisch, T. (2002) Cholesterol, essential fatty acids, and suicide. *Pharmacopsychiatry* **35(1)**, 1–5.
21. Guillemette, J., Hebert, M., Paquet, J., and Dumont, M. (1998) Natural bright light exposure in the summer and winter in subjects with and without complaints of seasonal mood variations. *Biol. Psychiatr.* **44(7)**, 622–628.
22. Graw, P., Recker, S., Sand, L., Krauchi, K., and Wirz-Justice, A. (1999) Winter and summer outdoor light exposure in women with and without seasonal affective disorder. *J. Affect. Disord.* **56(2–3)**, 163–169.
23. Blair-West, G.W., Mellsop, G.W., and Eyeson-Annan, M.L. (1997) Down-rating lifetime suicide risk in major depression. *Acta Psychiatr. Scand.* **95(3)**, 259–263.
24. Wattis, J.P. (1995) Difficulties in diagnosis of depression in the elderly. *Spectrum Int.* **XXXV(6–7)**.

## BIOSKETCHES

**Said Shahtahmasebi** is currently principal research methodology consultant and statistician, RaDiSol (R & D integrated Solutions), Christchurch, New Zealand. His work experiences are mainly in the United Kingdom and cover statistical and longitudinal modelling in a number of fields including public health, mental health, nursing, operational research, food, nutrition and gerontology. His area of interests and expertise are related to the longitudinal modelling of health related behaviour. He is the Founder of the Good Life Research Centre Trust in New Zealand, with the focus on holistic research for the benefit of the community.

**Shahnaz Shahtahmasebi** is a food and nutrition scientist and until recently was lecturing in nutrition and dietetics. She is currently providing consultation through her privately run clinic in Wagga Wagga, NSW Australia. Her research interests relate to nutritional behaviour and human health. She received her PhD in food science from Leeds University, U.K (1982).
